# Characterization of DNA Methylation Patterns and Mining of Epigenetic Markers During Genomic Reprogramming in SCNT Embryos

**DOI:** 10.3389/fcell.2020.570107

**Published:** 2020-09-02

**Authors:** Pengbo Cao, Hanshuang Li, Yongchun Zuo, Buhe Nashun

**Affiliations:** State Key Laboratory of Reproductive Regulation and Breeding of Grassland Livestock, School of Life Sciences, Inner Mongolia University, Hohhot, China

**Keywords:** DNA methylation, SCNT, WGCNA, epigenetic reprogramming, ZGA

## Abstract

Somatic cell nuclear transfer (SCNT), also known as somatic cell cloning, is a commonly used technique to study epigenetic reprogramming. Although SCNT has the advantages of being safe and able to obtain pluripotent cells, early developmental arrest happens in most SCNT embryos. Overcoming epigenetic barriers is currently the primary strategy for improving reprogramming efficiency and improving developmental rate in SCNT embryos. In this study, we analyzed DNA methylation profiles of *in vivo* fertilized embryos and SCNT embryos with different developmental fates. Overall DNA methylation level was higher in SCNT embryos during global de-methylation process compared to *in vivo* fertilized embryos. In addition, promoter region, first intron and 3′UTR were found to be the major genomic regions that were hyper-methylated in SCNT embryos. Surprisingly, we found the length of re-methylated region was directly related to the change of methylation level. Furthermore, a number of genes including Dppa2 and Dppa4 which are important for early zygotic genome activation (ZGA) were not properly activated in SCNT embryos. This study comprehensively analyzed genome-wide DNA methylation patterns in SCNT embryos and provided candidate target genes for improving efficiency of genomic reprogramming in SCNT embryos. Since SCNT technology has been widely used in agricultural and pastoral production, protection of endangered animals, and therapeutic cloning, the findings of this study have significant importance for all these fields.

## Introduction

Somatic cell nuclear transfer (SCNT) technology can not only restore pluripotency of mature somatic cells, but also generate new offspring by transplanting somatic cell nuclei into enucleated oocytes (Rodriguez-Osorio et al., 2012; Zuo et al., 2017). In 1952, Briggs and King performed the first cell reprogramming experiment in frog embryos (Briggs and King, 1952). Subsequently, Gurdon transplanted Xenopus laevis epithelial cells in tadpole stage into eggs with damaged nuclei. A small fraction of the eggs developed into normal embryos, demonstrating that differentiated somatic cells can still restore pluripotency (Gurdon, 1962). To date, SCNT has been successfully applied in variety of mammals to generate nuclear transferred embryos from the “Dolly sheep” to the macaque monkeys (Wilmut et al., 1997; Liu et al., 2018; Matoba and Zhang, 2018). Cloning with somatic cells enables replication of valuable animals which are endangered or with transgene (Keefer et al., 2002; Zuo et al., 2014). However, cloning efficiency is still very low in practical applications. For mice, only about 30% of SCNT embryos develop into blastocysts (Matoba et al., 2018). Accumulating evidences suggested that incomplete reprogramming of epigenetic status including chromatin accessibility, histone modifications and DNA methylation of the donor cell genome, is a determining factor for the low cloning efficiency (Cantone and Fisher, 2013; Niemann, 2016).

DNA Methylation is an important epigenetic modification that plays critical role in mammalian development (Wendy et al., 2010). DNA methylation is established and maintained by DNA methyltransferases (Dnmts) and can be de-methylated by Ten-Eleven-Translocation (Tet) protein-mediated oxidation process and thymine DNA glycosylase (TDG)-mediated base-excision repair pathway (Li et al., 1999; Gao et al., 2013; Kohli and Zhang, 2013; Liu et al., 2019). During pre-implantation development of the mice, both paternal and maternal genomic DNA undergoes extensive de-methylation reaching the lowest level by blastocyst stage. After fertilization, the paternal DNA is actively de-methylated, while the maternal DNA is passively de-methylated, and de-methylation happens faster in paternal genome than the maternal genome (Oswald et al., 2000; Fan et al., 2014). It has been reported that DNA methylation level of promoter region negatively correlate with gene expression during pre-implantation development and this negative correlation increase with time (Zhu et al., 2018). Studies in human embryos show that major wave of genome-wide de-methylation occurs in the 2-cell stage (Guo et al., 2014). Interestingly, evolutionary older repetitive elements de-methylate more than evolutionarily younger repetitive elements, suggesting that early embryos tend to retain higher residual DNA methylation on evolutionary younger and more active transposable elements (Li et al., 2015). In addition to the repetitive elements, genomic imprinting regions also exhibit methylation resistance. These regions maintain gametic DNA methylation patterns till adulthood, and deletion of the regions results in severe developmental defects (Surani et al., 1984; Mackay and Temple, 2017).

Global DNA de-methylation also happens in SCNT embryos, which is a critical step toward successful development (Nie et al., 2017). As early as 2001, Wendy et al. studied DNA methylation patterns in SCNT embryos by immunofluorescence staining and suggested that DNA methylation is incomplete in most SCNT embryos (Dean et al., 2001). In support of this view, subsequent studies confirmed that donor cell DNA methylation is not completely reprogrammed in SCNT embryos (Peat and Reik, 2012) and experimental efforts to overcome the anomalies in DNA methylation improved cloning efficiency. It has been reported that in the late 1-cell SCNT embryos, more than 20 genes (including: Rbm44, Sycp3, Tex19.1, Rpl39l, and Ccin, etc.) as well as long spreading elements (LINE) and long terminal repeats (LTR) were resistant to de-methylation (Chan et al., 2012). Meanwhile, re-methylation happens in some of the de-methylated DNA in SCNT embryos, which could negatively affect development efficiency (Gao et al., 2018). Therefore, methylation anomalies in SCNT embryos can be partially attributed to incomplete de-methylation or re-methylation of the donor somatic cell genome (Ma et al., 2014). Additionally, other epigenetic mechanism of pre-implantation and nuclear transferred embryos has been intensively explored and the findings have contributed to the improvement of cloning efficiency (Liu X. et al., 2016; Matoba et al., 2018; Wang et al., 2018; Bi et al., 2020).

In this study, we analyzed DNA methylation profiles of *in vivo* fertilized embryos and SCNT embryos which developed to different embryonic stages. We observed overall hyper-methylation in SCNT embryos, and further identified characteristics of abnormal methylation regions. Moreover, by combining DNA methylation and RNA-seq data and applying weighted gene co-expression network analysis (Langfelder and Horvath, 2008; Eckersley-Maslin et al., 2019), several key markers were found including Dppa2 and Dppa4 which are hyper-methylated and inactivated in nuclear transferred embryos. Our study suggested that abnormal methylation lead to failure in activation of certain critical genes for zygotic genome activation (ZGA) and may act as epigenetic barriers for genomic reprogramming in SCNT embryos. Understanding the underlying regulatory mechanisms of these epigenetic abnormalities will help us to design better strategy to overcome epigenetic barriers in genome reprogramming and increase efficiency of SCNT for animal cloning and pluripotent stem cell generation (Bao et al., 2019; Zhou et al., 2019; Sun et al., 2020).

## Materials and Methods

### Dataset Collection

The data used in this study was obtained from Gene Expression Omnibus (GEO) database. The single cell RNA-seq data was published by Shaorong Gao group in 2016, of which GEO number is GSE70605 (Liu W. et al., 2016). The DNA methylation data includes two parts: GSE98151 published by Shaorong Gao group in 2018 (Wang et al., 2018) and GSE108711 published by Yong Zhang group in 2018 (Gao et al., 2018). The two data sets covers SCNT embryos at different developmental stages: [zygote, 2-cell arrest (NA2), 2-cell to blastocyst (NB2), 4-cell arrest (NA4), 4-cell to blastocyst (NB4)]; *in vivo* fertilized 2-cell (WT2) and *in vivo* fertilized 4-cell (WT4); MII Oocyte and Cumulus cell (CC). For the RNA-seq data, each sample consists of 5 to 9 biological replicates. A pooled sample (30–50 embryos) was used for WGBS analysis. See also the Supplementary Tables S1 and S2.

### WGBS Data Analysis

For WGBS data processing, TrimGalore (v0.3.3) was used to remove adaptor and low-quality reads with parameter “–phred33–fastqc –paired –rrbs.” Then, the trimmed reads were mapped to the reference genome (mm9) with bsmap (v2.89). Mcall was used to calculated methylation level of each CpG sites (Liu W. et al., 2016). The CpG sites with coverage less than three reads were discarded (Gao et al., 2018).

In differential DNA methylation analysis, a site with |Δβ| >0.2 was defined as a differential DNA methylation site. DNA methylation level of the gene was represented by average DNA methylation level of the CpG sites in gene promoter (Gao et al., 2018). SMART2 package of Python was used for differential methylation region analysis (Differentially Methylated Regions, DMRs). The parameters were set to CpG Distance: 500, AbsMeanMethDiffer: 0.3, p_DMR: 0.05 (Liu et al., 2015). The rescue score (RS) defined from Gao et al. (2018) was used to identify three different DNA methylation patterns of DMRs (pDMRs: persistently methylated differential methylated regions, dDMRs:de-methylated differential methylated regions and rDMRs: re-methylated differential methylated regions) in 2 and 4-cell stages, the formula is as follows (for example in 2-cell stage):

(1) RS=abs(MethylNTembryos-MethylWTembryos)-λ×abs(MethylNT 2-cell-MethylWTembryos)

where Methyl indicates methylation levels, and detail parameter setting can be found in Gao’s article (Gao et al., 2018).

### RNA-Seq Data Analysis

RNA-seq raw data was first converted from the sra format to the fastq format using SRA-Toolkits. Then, quality-controlled FastQC was used to eliminate low-quality data^1^. All valid reads were mapped to the mouse reference genome (mm9) based on the principle of base-pair pairing, using the software Hisat2 (Pertea et al., 2016) (v 2.1.0). Stringtie (Pertea et al., 2015, 2016) (v 1.3.3) and Ballgown (Pertea et al., 2016) were used for sequence assembly and transcript quantification. Expression levels of each gene were quantified with fragments per kilobase of exon model per million mapped reads (FPKM) (Matoba et al., 2014).

All genes with FPKM < 0.1 in the duplicate samples were removed and the remaining genes were considered as expressed genes (Cao et al., 2014). R package DEseq2 (Love et al., 2014) was used in differentially expressed genes (DEGs) analysis. Genes with absolute log2 fold change >1 and *q*-value < 0.05 were defined as DEGs, where *q*-value is the result of *P*-value correction (Liu W. et al., 2016). The gene expression values were standardized by logarithmic transformation for further study. The standardized formula is as follows:

$$x^{{}^{*}}=\log_{10}\left(x+1\right)$$

### Weighted Gene Co-expression Network Analysis

Through combined analysis of whole-genome bisulfite sequencing (WGBS) and RNA-seq data, we obtained genes that were down-regulated in gene expression level affected by high DNA methylation in SCNT embryos. In order to identify hub epigenetic markers, the weighted gene co-expression network (WGCNA) approach was used to construct a co-expression network (Langfelder and Horvath, 2008; Zuo et al., 2016). Firstly, we constructed the adjacency matrix: *a*_ij_ = cor(*x*_i_,*x*_j_)^β^. *x*_i_, *x*_j_ represent the expression values of the two genes, respectively. And the parameter β is the weighted coefficiency of the matrix. The soft threshold (β) was identified base on pickSoft Threshold function with default parameters setting (Supplementary Figure S5A). The threshold (β) for adjacency matrix is set to five, making the network close to non-scale. The hierarchical clustering algorithm (Mallik and Zhao, 2019), hclust (*d*, method = “average”) was performed to identify the similarity among the samples with different developmental fates (Supplementary Figure S4B). Next, the blockwiseModules function was implemented using the following parameters: power 5, minModuleSize 30, mergeCutHeight 0.25 to detect modules. The FPKM of genes from *in vivo* fertilized embryos and SCNT embryos which developed to 2-cell or 4-cell stages was used as expression value. The different types of embryo classification variables in different periods were converted into form of a 0–1 matrix (1 indicates that it belongs to this group or has this attribute, 0 indicates that it does not belong to this group or does not have this attribute). In addition, the expression of the methyltransferases (Dnmt1, Dnmt3a, Dnmt3b), and the demethylases (Tet1, Tet2, and Tet3) from *in vivo* fertilized embryos were used together as the phenotypic matrix to identify genes which are closely related to changes in DNA methylation in cell reprogramming.

### Gene Ontology and KEGG Pathway Analysis

Functional annotation was performed with the Database for Annotation, Visualization and Integrated Discovery (DAVID) Bioinformatics Resource^2^. Gene ontology terms and KEGG pathways for each function cluster were summarized to a representative term and *P*-values were plotted to show the significance.

### Statistical Analysis

R programming language including the R/Biocoductor software packages^3^ was mainly used in the statistical analysis and data visualization. Program packages used for data processing consisted of Python package SMART2 and R packages (ChiPseeker, dplyr, psych etc.). The gene co-expression network was constructed by the software Cytoscape. Package MCODE was used for module mining from the network. The correlation between gene expression and DNA methylation was calculated with Spearman correlation coefficient. Most of the data visualization in this study was completed by R packages (ggplot2, UpSetR, gcookbook, CMplot etc.).

## Results

### Abnormal High DNA Methylation Levels Were Observed Across Whole Genome Region in SCNT Embryos

Somatic cell nuclear transfer technology has been widely used in animal cloning and generation of pluripotent embryonic stem cells. However, early embryonic arrest happens in most SCNT embryos. Accumulating evidence suggests that abnormal DNA methylation is one of the main contributing factors for the low developmental rate of SCNT embryos (Peat and Reik, 2012). In order to have a deeper insight into the relationship between DNA methylation and low developmental potential of SCNT embryos, we conducted genome-wide DNA methylation analysis based on whole-genome bisulfite sequencing (WGBS) data obtained from different developmental stages of SCNT embryos, including: zygote, 2-cell arrest (NA2), 2-cell to blastocyst (NB2), 4-cell arrest (NA4), 4-cell to blastocyst (NB4); and *in vivo* fertilized embryos including: *in vivo* fertilized 2-cell (WT2), *in vivo* fertilized 4-cell (WT4); and Cumulus cell (CC) (Gao et al., 2018).

**FIGURE 1:**
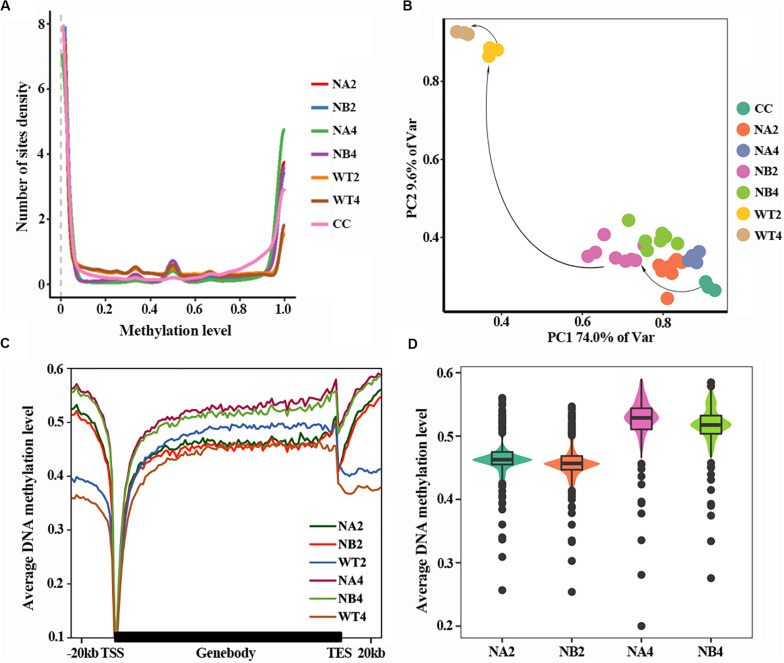
Aberrant DNA methylation occurs in SCNT embryos. **(A)** DNA methylation density distribution of different types of embryos. Higher probability density distribution of hyper-methylated regions was observed in nuclear transferred embryos. **(B)** PCA plot of DNA methylation from all samples used in this study. Different colors represent different samples indicated in the right. **(C)** Average DNA methylation levels were determined along the gene bodies, mm9 reference genome were split to 100 bins with equal proportion, the upstream and downstream 20 kb of the genes were split into 20 bins with the length of 1 kb. Methylation levels were average methylation levels on 1 bin. **(D)** Violinplot of DNA methylation levels of SCNT embryos at 2-cell and 4-cell stages.

DNA methylation density distribution of different types of cells showed different patterns (Figure 1A). Overall, cells of all types displayed bipolar methylation density distribution. Of note, the SCNT embryos showed higher probability density distribution in hyper-methylated regions than *in vivo* fertilized embryos, suggesting that embryos with different developmental fate have different DNA methylation patterns.

In order to take a closer look at the DNA methylation dynamics of the SCNT and *in vivo* fertilized embryos, principal component analysis (PCA) was performed (Figure 1B). SCNT and *in vivo* fertilized embryos split into two separate clusters in the PCA map, but nuclear transferred embryos that were arrested at early stages (NA) or developed to blastocyst (NB) were not clearly separated. Furthermore, all embryos at different developmental stages spread along the PC1 axis according to the methylation level. This indicated that DNA methylation level was significantly different between SCNT and fertilized embryos, but not in embryos at different stages or with different developmental fates.

Then we used a higher resolution scale to examine the differences in DNA methylation levels of different embryos at genome wide level. Relative position of the genome was divided in 100 bins and average DNA methylation level of these positions was counted (Figure 1C). Overall, DNA methylation level of all embryo types reached the lowest point (about 0.1) at transcription start site (TSS). The overall methylation level of SCNT embryos was higher than that of *in vivo* fertilized embryos, especially at the 4-cell stage. Furthermore, comparing methylation levels of SCNT embryos arrested at early stages (NA2, NA4) with those developed to blastocyst (NB2, NB4), higher DNA methylation levels were observed across the whole genome region in SCNT embryos that were arrested at early stages (Figures 1C,D). Consistent with previous report that impaired DNA methylation can be an unavoidable barrier in cloned embryos (Gao et al., 2018), these findings indicated that the abnormal DNA de-methylation of nuclear transferred embryos may closely relate to the early development arrest of nuclear transferred embryos.

### Characterization of Aberrant Differential Methylation Region (DMRs) in SCNT Embryos

**FIGURE 2:**
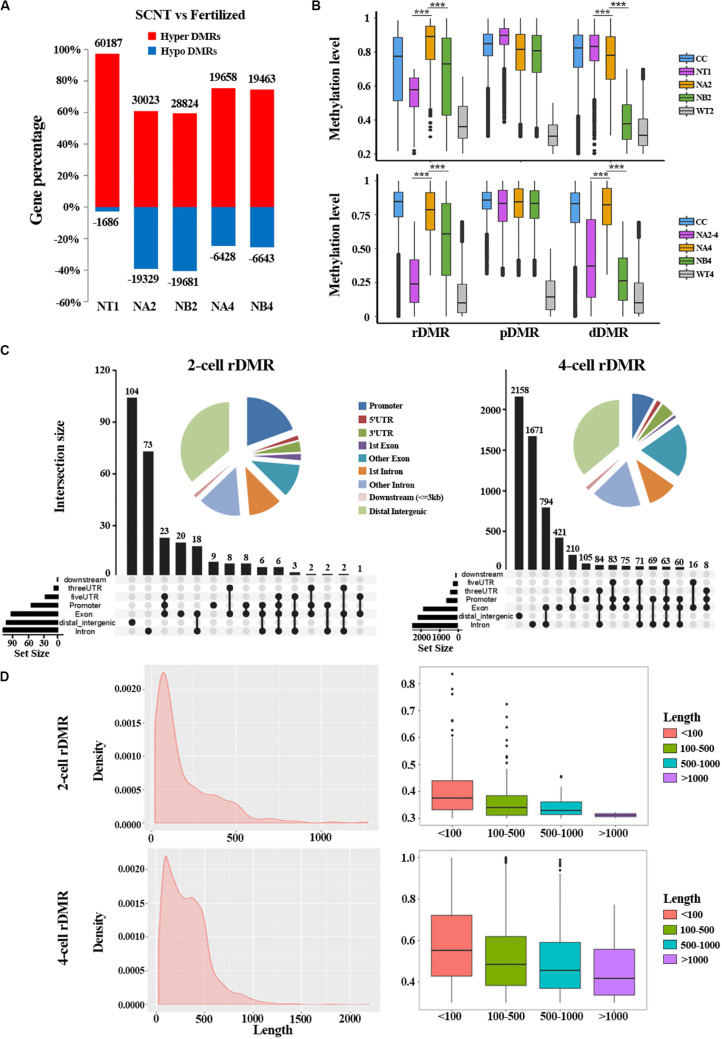
Aberrant DNA methylation patterns revealed by differential DNA methylation region analysis in SCNT embryos. **(A)** Barplot of DMRs in the SCNT and in vivo fertilized samples at different developmental stages. The red and blue bars represent hyper-DMRs and hypo-DMRs, respectively. **(B)** Boxplot of hyper DMRs in SCNT and *in vivo* fertilized embryos. Each box represents a hyper DMR, and colors represent different types of embryos at different development stage. DMRs were classified into three categories: dDMRs (de-methylated differential methylated regions); rDMR (re-methylated differential methylated regions); and pDMR (persistent differential methylated regions). Differences are statistically significant. *P-value < 0.05; **P-value < 0.01; ***P-value < 0.001, *t*-test. **(C)** Genomic distribution of rDMRs. Pie charts represent proportion of rDMRs in different genomic contexts. Inset graphs represent number of DMRs distributed in single or combined genomic regions. **(D)** Probability density distribution of rDMRs in 2-cell and 4-cell stage (left). Box plots show change in DNA methylation level within different length of rDMRs (right panel, less than 100 bp, between 100 and 500 bp, between 500 and 1000 bp, and greater than 1000 bp).

To further study the underlying molecular mechanism of the abnormal DNA methylation in SCNT embryos, we performed differential methylation region (DMR) analysis on SCNT embryos and *in vivo* fertilized embryos using python packages “SMART2.” The identified DMRs were divided into Hyper DMRs and Hypo DMRs by comparing DNA methylation levels of the DMRs in SCNT and *in vivo* fertilized embryos (Hyper DMRs: DNA methylation levels _(SCNT embryos – fertilized embryos)_ > 0.2) (Figure 2A). Consistent with previous studies from Gao’s group (Gao et al., 2018), these Hyper DMRs show three different methylation types (persistently methylated differential methylated regions: pDMRs, de-methylated differential methylated regions: dDMRs and re-methylated differential methylated regions: rDMRs) in 2-cell and 4-cell embryos with different development fate (Figure 2B).

In order to investigate whether the distribution of these three types of DMRs has genome regional preference, we mapped these DMRs to different genomic regions and found that rDMRs were mainly located in distal intergenic region, promoters, other introns, first introns and other exons. It is worth noticing that the 3′UTR region also had a relatively high proportion of rDMRs distribution (Figure 2C). Similar distribution pattern was also observed for pDMRs and dDMRs (Supplementary Figures S1A,B). These results indicated that distribution of DMRs have regional preferences, in which the promoter and the first intron are main functional regions enriched with DMRs. Chromosome preference analysis showed that the 13, 14, and 15 chromosomes had significantly less rDMRs, while other chromosomes were enriched in rDMRs (Supplementary Figure S1C). Similar results were also observed both for dDMRs and pDMRs (Supplementary Figure S1C). We speculate that there may be certain mechanisms for 13, 14, and 15 chromosomes to escape from abnormal DNA methylation.

Moreover, we found that the rDMRs at the 2-cell and 4-cell stage were mainly enriched in the region about 100 to 500 bp (Figure 2D), indicating that most rDMRs are short in length (<500 bp). Surprisingly, the methylation level was correlated with the length of DMRs, in which the rDMRs less than 100 bp had the most changes in DNA methylation level. With the increase of the length of rDMRs, the change in DNA methylation level decreased gradually (Figure 2D, right). Similar results were obtained both for dDMRs and pDMRs (Supplementary Figure S2). Taken together, these results indicated that hyper DMRs tend to appear in shorter regions, and these short DMRs had greater changes in DNA methylation levels than long DMRs.

### Hyper-DMRs Regulate Important Biological Processes

**FIGURE 3:**
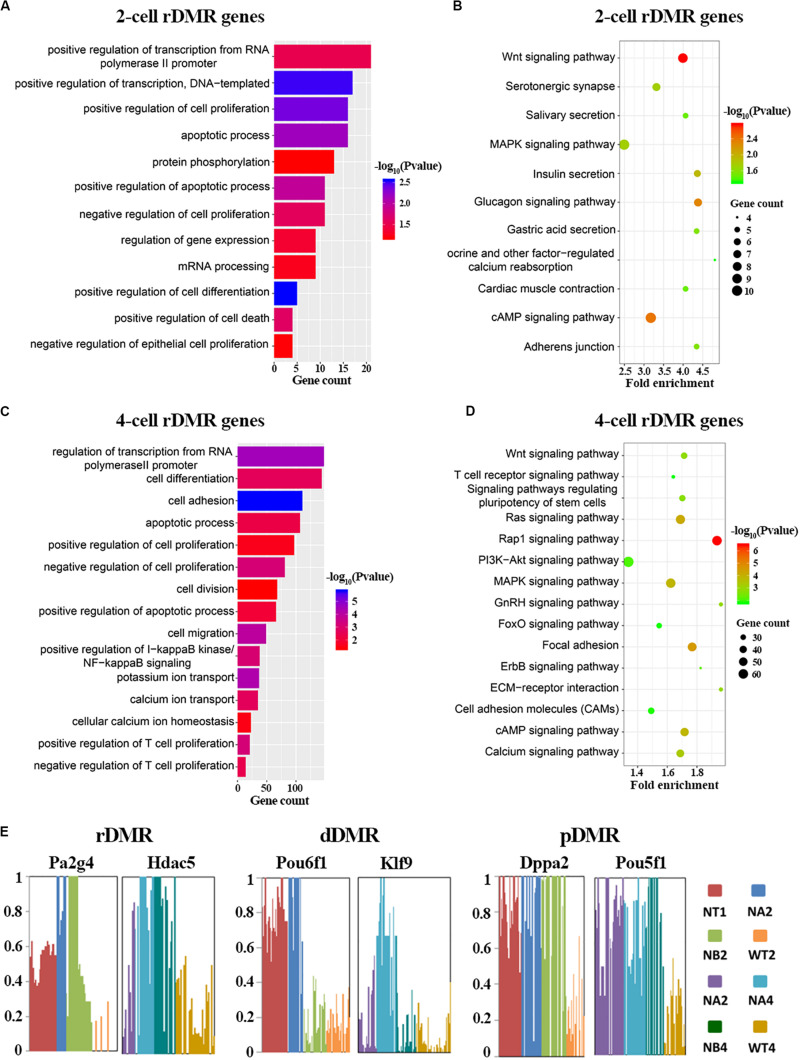
Functional analysis of rDMRs. **(A,C)** GO term analysis of rDMRs in 2-cell stage and 4-cell stage, respectively. **(B,D)** KEGG pathway enrichment of rDMRs in 2-cell stage and 4-cell stage, respectively. **(E)** Examples of genes with different DMR patterns. Different samples were represented by different bar colors. Ordinate represents DNA methylation level.

Having described properties of the abnormal methylation patterns in SCNT embryos, we set out to explore the biological functions of these Hyper-DMRs. We mapped these DMRs to genes, and performed GO and KEGG enrichment analysis (Figures 3A–D and Supplementary Figure S3). In consistent with the active DNA replication and cell proliferation in early pre-implantation embryo, GO term analysis (Figures 3A,C) showed that rDMRs were mainly enriched in genes that are related to positive regulation of transcription, positive regulation of cell proliferation, and positive regulation of transcription from RNA polymerase II promoter. In sharp contrast to above findings, genes that are related to apoptosis process and positive regulation of cell death were also enriched in the GO term analysis, indicating that these cellular processes might safeguard cell reprogramming to be proceeded in right direction. However, in SCNT embryos, the hyper methylation of the rDMRs could inhibit expression of the genes involved in above mentioned biological processes (Spearman rank correlation coefficient, ρ = −0.53; Supplementary Figure S4A), leading to inadequate genomic reprogramming and early embryonic arrest in SCNT embryos, which is consistent with findings in other studies (Gao et al., 2018). In the KEGG enrichment analysis (Figures 3B,D), we found that rDMRs were mainly involved in Wnt signaling pathway, cAMP signaling pathway, and MAPK signaling pathway. These pathways are important for regulation of cell reprogramming and carcinogenesis. In addition, rDMRs were also enriched in pathways such as cell adhesion and calcium ion reabsorption, which are also important pathways for cell reprogramming. It has been reported that cell adhesion affects the Hippo pathway and damages Yap localization, thereby limits transcription of Tead4 and impairs activation of key genes for cell reprogramming (Shi et al., 2017). GO term analysis and KEGG enrichment analysis showed that dDMRs and pDMRs were also enriched in genes that are important for cellular reprogramming (Supplementary Figure S3). Taken together, these findings suggested that hyper methylation of DMRs in SCNT embryos could inhibit expression of genes involved in important cellular pathways and lead to early developmental arrest in SCNT embryos.

In order to further understand the role of hyper-DMRs in regulating genomic reprogramming in SCNT embryos, we screened the genes in rDMRs, dDMRs and pDMRs categories, respectively. We found several genes with important functions in development, including Pa2g4, Hdac5, Klf9, Dppa2, Pou5f1 (Figure 3E). Hdac5 is a histone deacetylase involved in the regulation of early embryo development. Klf9 and Pou5f1 are key transcription factors that play important roles in early embryonic development (Chen et al., 2016; Li et al., 2019). These genes were all hyper-methylated in SCNT embryos compared to *in vivo* fertilized embryos and showed highly specific differential methylation patterns. Taken together, it is likely that hyper-DMRs in SCNT embryos directly impair expression of certain developmentally important genes, and paly detrimental effect on early embryonic development.

### Activation of Key Genes in SCNT Embryos Was Impaired by DNA Hyper-Methylation

It has been widely accepted that DNA methylation of promoter region negatively regulates gene expression. Thus, it is possible that hyper-methylation in SCNT embryos prevents timely activation of certain genes that are critical for genomic reprogramming and embryonic development in the SCNT embryos. To further verify this hypothesis, we first analyzed transcriptome profile of *in vivo* fertilized embryos. Oocyte genes were mostly silent, but many genes were activated from zygote to the 2-cell stage during the zygotic genome activation (ZGA). By the 4-cell stage, genes continue to be activated, but at the same time, some genes that were active in previous phase were silenced, showing a cell-phase-specific pattern (Supplementary Figure 4B group2). The GO term analysis showed that these genes that were inactivated at 4-cell stage were mainly enriched in cellular processes such as transcription, cell proliferation, and cell cycle.

Next, we performed an integrated analysis of RNA-seq data and DNA methylation data to further identify genes whose expression was influenced by aberrant DNA methylation in SCNT embryos. Based on the classification standard in previous studies (Gao et al., 2018), all genes were divided into three different categories: up-regulated genes with decreased methylation levels (2-cell *n* = 77; 4-cell *n* = 57), down-regulated genes with increased methylation levels (2-cell *n* = 213; 4-cell *n* = 300) and other genes whose expression was independent of methylation levels. Interestingly, there were 142 down-regulated ZGA genes with increased methylation levels in NT-arrested 2-cell stage embryo, suggesting that the failed activation of these genes was caused by hyper-methylation in the nuclear transferred embryos.

Moreover, Heatmap analysis also showed that strong negative correlation exists between the expression and methylation levels of these genes both in the *in vivo* fertilized and NT-arrested 2-cell embryos (Supplementary Figure S4C), further indicating that incomplete activation of these ZGA related genes was due to the hyper-methylation in the SCNT embryos. In order to dissect functional roles of the genes, we had a closer look and found that this set of genes included a number of genes that are known to be important for cellular reprogramming. Among them, Kdm5b, Zscan5b, and members of the zinc finger family proteins (Zfp217, Zfp574, Zfp662, Zfp809, and Zfp868) all work as reprogramming promotion factors. Tead4, Obox6, and Yy1 are key transcription factors involved in regulation of pluripotency (Sakurai et al., 2017; Wu et al., 2017; Hu et al., 2019; Schiebinger et al., 2019). Dppa4 is a pluripotency-related factor during early development (Hernandez et al., 2018). The polycomb protein Pcgf5 co-localizes with active histone marks such as H3K27ac and H3K4me3, and associate with highly expressed genes (Yao et al., 2018). Thus, our analysis identified a number of developmentally important genes whose activation or expression was impaired by DNA hyper-methylation in the SCNT embryos.

### Identification of Candidate Hub Marker Genes in SCNT Embryos by Weighted Gene Co-expression Network Analysis

**FIGURE 4:**
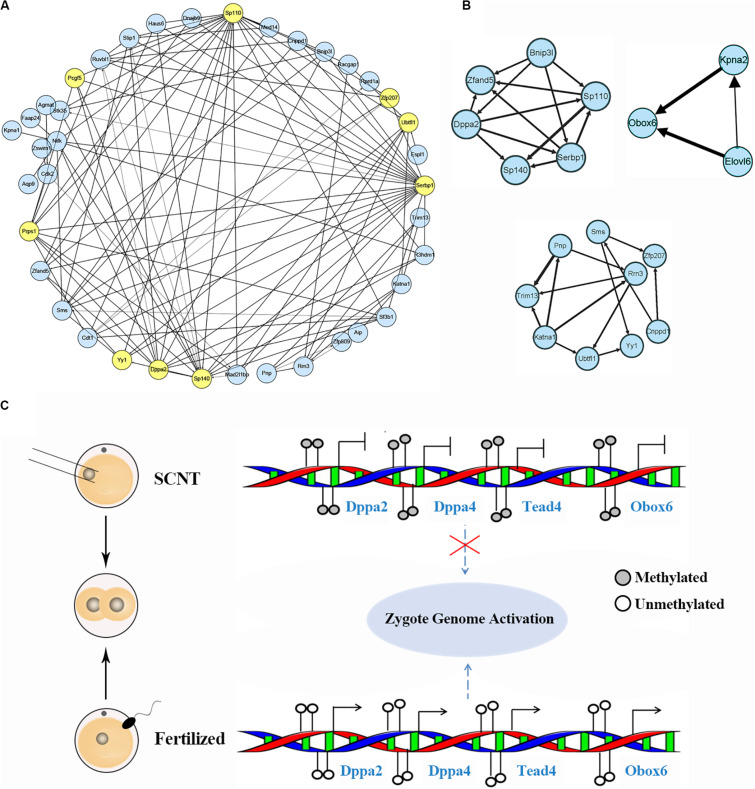
Identification of hub marker genes in SCNT embryos. **(A)** Network diagram showing interaction of genes in the two modules of WGCNA production. Hub node genes are highlighted by yellow. **(B)** Co-expression network of key genes. The genes in the three networks are highly connected in the networks calculated by module mining. **(C)** Promoter DNA methylation of certain transcription factors like Dapp2/Dapp4 leads to failed zygotic genome activation in SCNT embryos, while de-methylated promoter of these genes in fertilized embryo safeguard proper activation of zygote genome. Filled circle represents DNA methylation, open circle represents DNA de-methylation.

Based on aforementioned analysis, we obtained a set of genes down-regulated by increased DNA methylation in SCNT 2-cell and 4-cell embryos, whose timely activation and expression are likely to be required for proper genomic reprogramming. In order to further explore hub marker genes, we conducted weighted gene co-expression network analysis using R package “WGCNA.” The threshold (β) for adjacency matrix was set to five, making the network close to non-scale (Supplementary Figure S5A). In module clustering, we got three modules ME0, ME1, and ME2. Since ME1 and ME2 were clustered closely (Supplementary Figure S5C), the genes in these two modules were used to construct co-expression networks, and key nodes in the networks were selected as candidate reprogramming barrier markers (Figure 4A and Supplementary Figure S4D). Based on topological properties of the networks, we labeled genes with high degree of node (hub node genes) and identified candidate genes including Ago2, Sp110, Sp140, Pcgf5, Zfp207, Ubtfl1, Dppa2, Dppa4, Yy1, Klf2, Klf9, Tead4. Among these genes, Dppa2 and Dppa4 are known as important pluripotency factors during early embryonic development, both of which affect ZGA by regulating Dux (Eckersley-Maslin et al., 2019). Yy1 is a key transcription factor for pluripotency genes activation (Chen et al., 2017), and Zfp207 is a zinc finger protein that plays a key role in pluripotency maintenance. Klf2 and Klf9 are members of the same pluripotent transcription factor Klf4 family (Martin et al., 2001). Tead4 is a key transcription factor that participates in pluripotency regulation. Thus, our weighted gene co-expression network analysis identified a set of candidate marker genes that play important roles in genomic reprogramming.

Then, we performed module mining using the Cytoscape enhancement package “MCODE” to find highly correlated eigengenes in the two networks (Figure 4B). Three small modules composed of highly connected eigengenes were found, which included Ubtfl1, Sp110, Sp140, Serbp1, Dppa2, Yy1, and Zfp207. Some of these genes have been reported to play key roles in cell reprogramming. Among them, Rrn3 is a specific RNA polymerase transcription initiation factor and necessary for mammalian RNA polymerase formation (Herdman et al., 2017). Obox6 is a key transcription factor that enhances reprogramming efficiency (Li et al., 2020). Taken together, our WGCNA analysis identified a number of developmental key genes whose expression was impaired in SCNT embryos probably due to hyper DNA methylation, further suggesting that these genes may act as epigenetic barrier during genomic reprogramming in SCNT embryos (Figure 4C).

## Discussion

Abnormal DNA methylation has been considered to be an important factor affecting reprogramming and developmental efficiency of SCNT embryos (Dean et al., 2001; Peat and Reik, 2012). In this work, we found that overall DNA methylation level was higher in SCNT embryos during global de-methylation process compared to *in vivo* fertilized embryos. In addition, integrated analysis of DNA methylation and transcriptome data identified a set of genes that were down-regulated by hyper DNA methylation in SCNT embryos. WGCNA analysis further identified several candidate epigenetic hub marker genes critical for successful genomic reprogramming. These findings shed more light on how DNA methylation regulates gene expression and genomic reprogramming in the SCNT embryos.

The three abnormal DNA methylation patterns (rDMRs, pDMRs, dDMRs) identified in SCNT embryos all locate in key functional genomic regions and regulate important biological processes during development. In consistent with previous studies (Gao et al., 2018), we also found that DNA methylation level of rDMR negatively correlates with gene expression, indicating that rDMRs could inhibit gene expression in SCNT embryos. Interestingly, shorter DMRs showed greater changes in methylation level.

Through weighted gene co-expression network analysis, we identified a set of candidate marker genes that play important roles in genomic reprogramming. These marker genes are important for early zygotic gene activation, but not properly activated in SCNT embryos, suggesting that incomplete activation of these genes may act as epigenetic barriers during genomic reprogramming in SCNT embryos. In our work, we found both Dppa2 and Dppa4 were hyper-methylated and barely expressed in SCNT embryos. Since both of them are important for pluripotency maintenance and act upstream of Dux, inadequate expression of these two genes could lead to downstream Dux unexpressed. In support of this view, transcriptome analysis found little or no expression of Dux in SCNT 2-cell embryos. Accumulating evidence suggest that Dux is highly expressed in 2-cell like cell and play a key role in genomic reprogramming and ZGA activation (Chen and Zhang, 2019; Eckersley-Maslin et al., 2019; Guo et al., 2019). Thus, it is possible that inactivation of Dux results in incomplete ZGA and lead to early developmental arrest in SCNT embryos.

Similarly, Obox6 and Tead4 are key transcription factors that are able to promote genomic reprogramming (Sakurai et al., 2017; Schiebinger et al., 2019). However, both of them are hyper-methylated in SCNT embryos and expressed lowly. Thus, their inadequate expression may also contribute to the low reprogramming and developmental efficiency of SCNT embryos. Studies have shown that both H3K9me3 and H3K27me3 act as epigenetic barriers for SCNT reprogramming, and overexpression of their de-methylases Kdm4b and Kdm4d could improve reprogramming efficiency (Liu W. et al., 2016; Matoba et al., 2018). Thereby, it is possible that experimental ways to induce de-methylation of candidate barrier genes (such as Dapp2, Dapp4, Obox6, Tead4) or directly compensating their expression in SCNT embryo could improve efficiency of genomic reprogramming and overcome early embryonic arrest in SCNT embryos (Eckersley-Maslin et al., 2020; Gretarsson and Hackett, 2020).

In conclusion, we did comprehensive analysis of genome wide DNA methylation in SCNT and *in vitro* fertilized embryos and deepened our understanding of the aberrant DNA methylation in nuclear transfer embryos. It has been demonstrated by Gao’s group and Zhang’s group that incomplete DNA methylation reprogramming, especially the DNA re-methylation, hinders the developmental potential of nuclear transferred embryos (Liu W. et al., 2016; Gao et al., 2018). Based on these studies, we further explored the abnormal DNA methylation in SCNT embryos and found that re-methylation was more likely to occur in promoter, first intron and 3′UTR regions. Interestingly, shorter DMRs had greater changes in methylation level and importantly, we provided several candidate target genes for overcoming epigenetic barrier in SCNT embryos including: Dppa2, Dppa4, Obox6 and Tead4. Our findings could shed more light on the underlying mechanism of how hyper-methylation negatively regulates gene expression and genomic reprogramming. The findings in this study provided valuable reference for conquering reprogramming obstacle of SCNT and has potential to improve cloning efficiency in practical applications (Onishi et al., 2000; Yan et al., 2020; Zhao et al., 2020). However, our findings are based on genome wide data analysis and need further experimental validation. The candidate genes should be overexpressed in SCNT embryos to see if the compensation of these genes could improve reprogramming efficiency. In addition, the mechanism of DNA re-methylation which happens during SCNT embryonic development need to be further explored.

## Data Availability Statement

All datasets presented in this study are included in the article/Supplementary Material.

## Author Contributions

YZ and BN conceived the study. PC and HL performed the bioinformatics analysis. BN wrote the manuscript with assistance from PC, YZ, and HL. All authors contributed to the article and approved the submitted version.

## Conflict of Interest

The authors declare that the research was conducted in the absence of any commercial or financial relationships that could be construed as a potential conflict of interest.

## Footnotes

1 http://www.bioinformatics.babraham.ac.uk/projects/fastqc/

2 https://david.ncifcrf.gov/home.jsp

3 http://www.bioconductor.org
