# Supplementary material for: Characterization of DNA Methylation Patterns and Mining of Epigenetic Markers During Genomic Reprogramming in SCNT Embryos
Source: Front Cell Dev Biol. 2020 Sep 2;8:570107. doi: 10.3389/fcell.2020.570107 (PMC7492385; doi:10.3389/fcell.2020.570107)
Supplement: Supplementary file 1 [file Data_Sheet_1.pdf]

Supplementary material

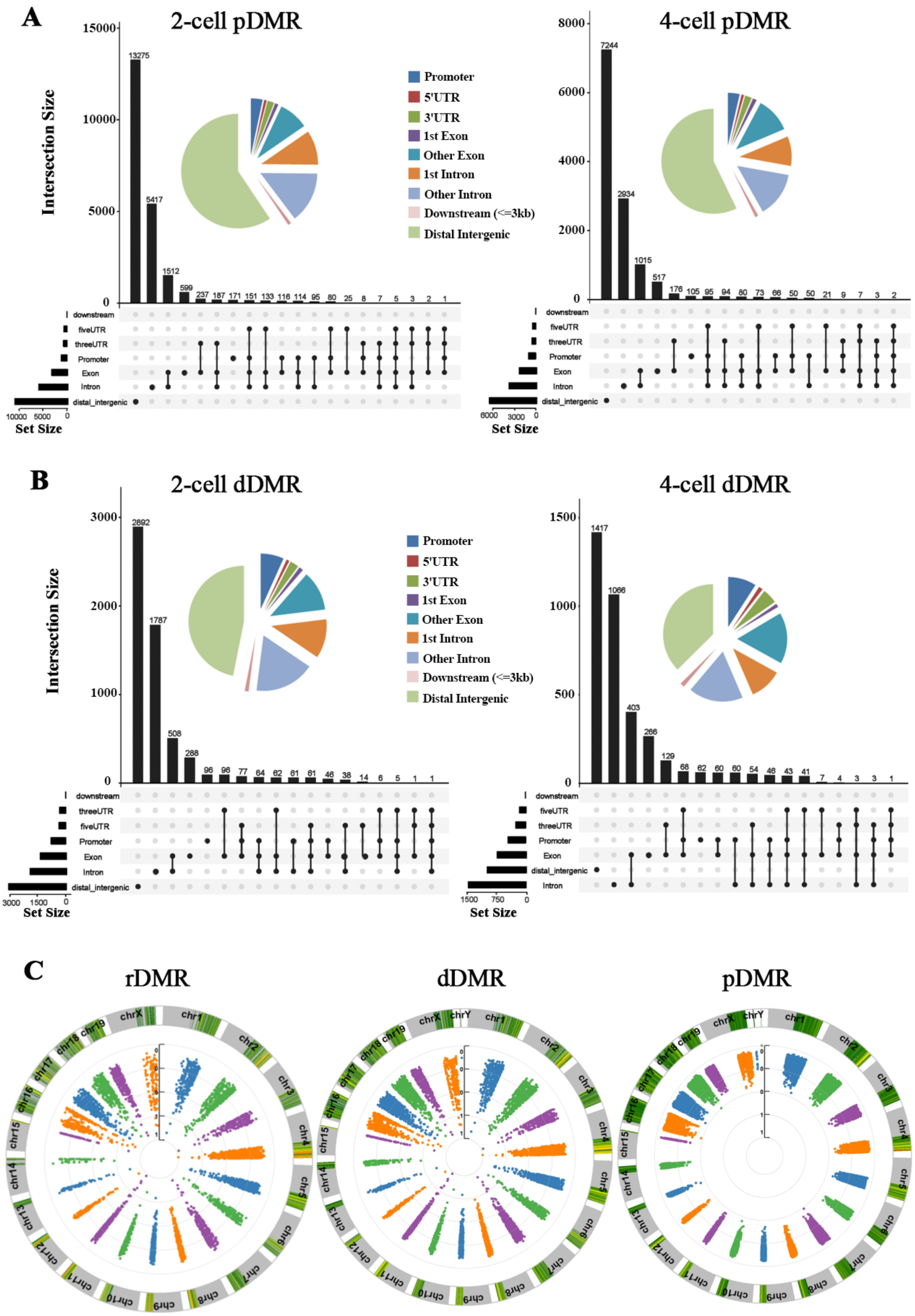

Supplementary Figure 1 Distribution of DMRs genome functional areas and chromosomes.

(A,B) Genome functional areas distribution of pDMRs and dDMRs. Pie charts represent distribution proportion of DMRs in different genomic regions, and the inset graphs represent the number of DMRs distributed in single or combined genomic regions.

(C) Chromosome preference analysis of rDMRs, pDMRs and dDMRs. The number of dots represents the DMRs distribution across different chromosomes.

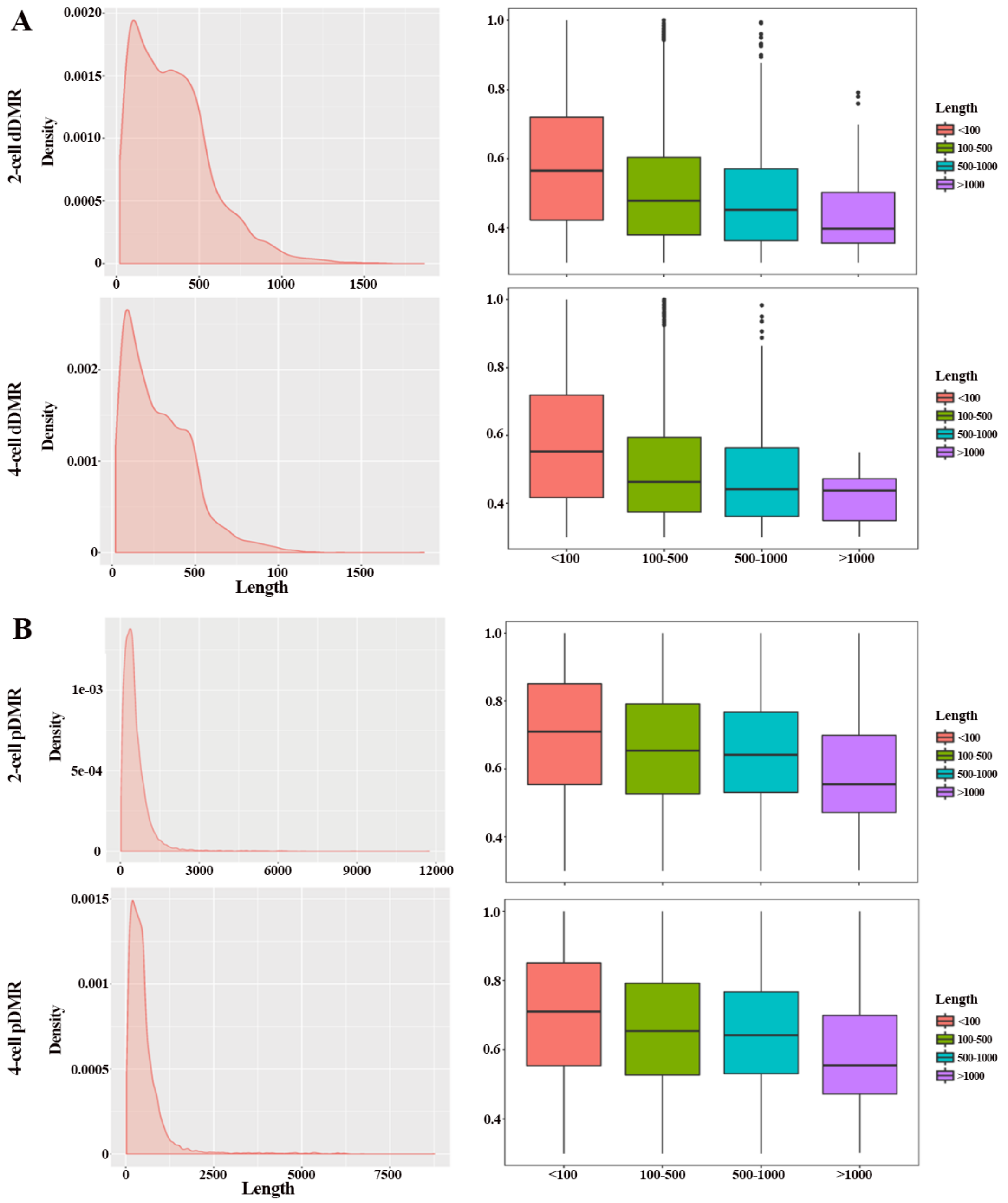

Supplementary Figure 2 Relationship between the length of DMRs and the change in DNA methylation level.

(A,B) Probability density distribution of the length of dDMRs and pDMRs (left) and box plots (right) showing the change of DNA methylation level within different region length (less than 100bp, between 100bp - 500bp, between 500bp - 1000bp, and greater than 1000bp) of DMRs (dDMRs and pDMRs) (right).

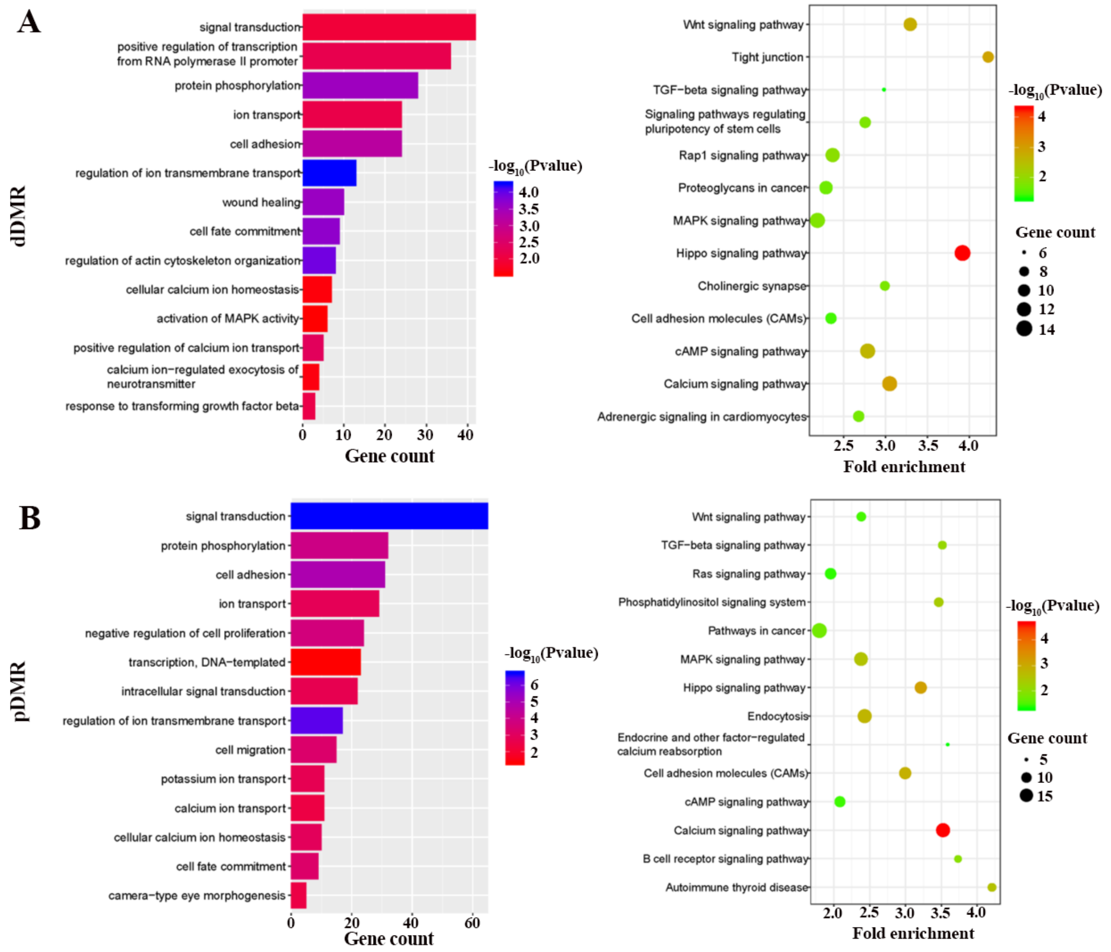

Supplementary Figure 3 Functional analysis of dDMR and pDMR.
(A) GO term analysis of dDMRs (left) and KEGG pathway enrichment of dDMR (right).
(B) GO term analysis of pDMRs (left) and KEGG pathway enrichment of pDMR (right).

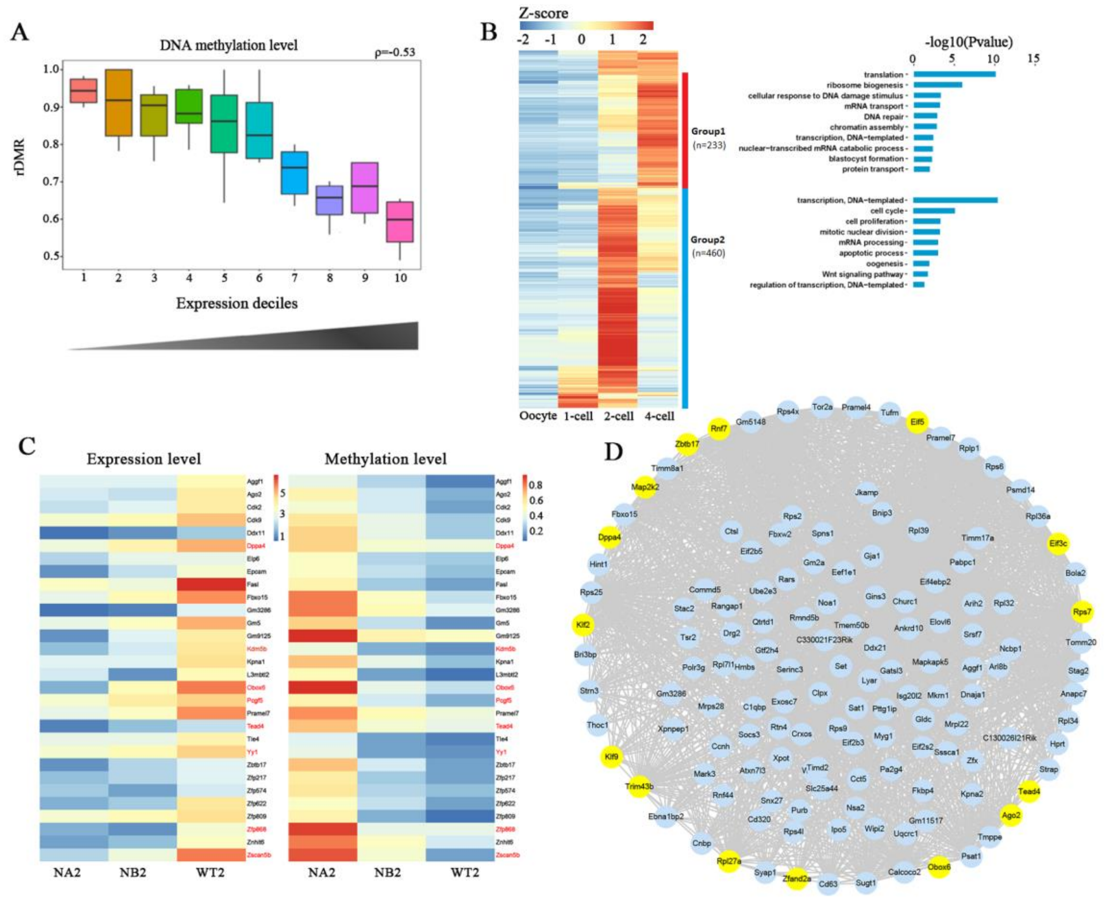

Supplementary Figure 4 ZGA related genes with hyper methylation levels failed to activate in clone arrest embryos.

(A) Correlation between methylation level of rDMR and gene expression. In violin plots, the DNA methylation in genes promoter are divided into 10 deciles based on gene expression, Spearman coefficient ρ is -0.53.

(B) The heat map (left) of genes expression in fertilized embryos. Functional enrichment analysis (right) shows the biological process of totipotency-related genes expressed at 2-cell stage (group2) and 4-cell stage (group1).

(C) Heatmaps showing the expression levels (left) and DNA methylation levels (right) of 30 2-cell genes affected by hypermethylation patterns.

(D) Network diagram showing interaction of genes in the two modules of WGCNA production. Hub node genes are highlighted by yellow.

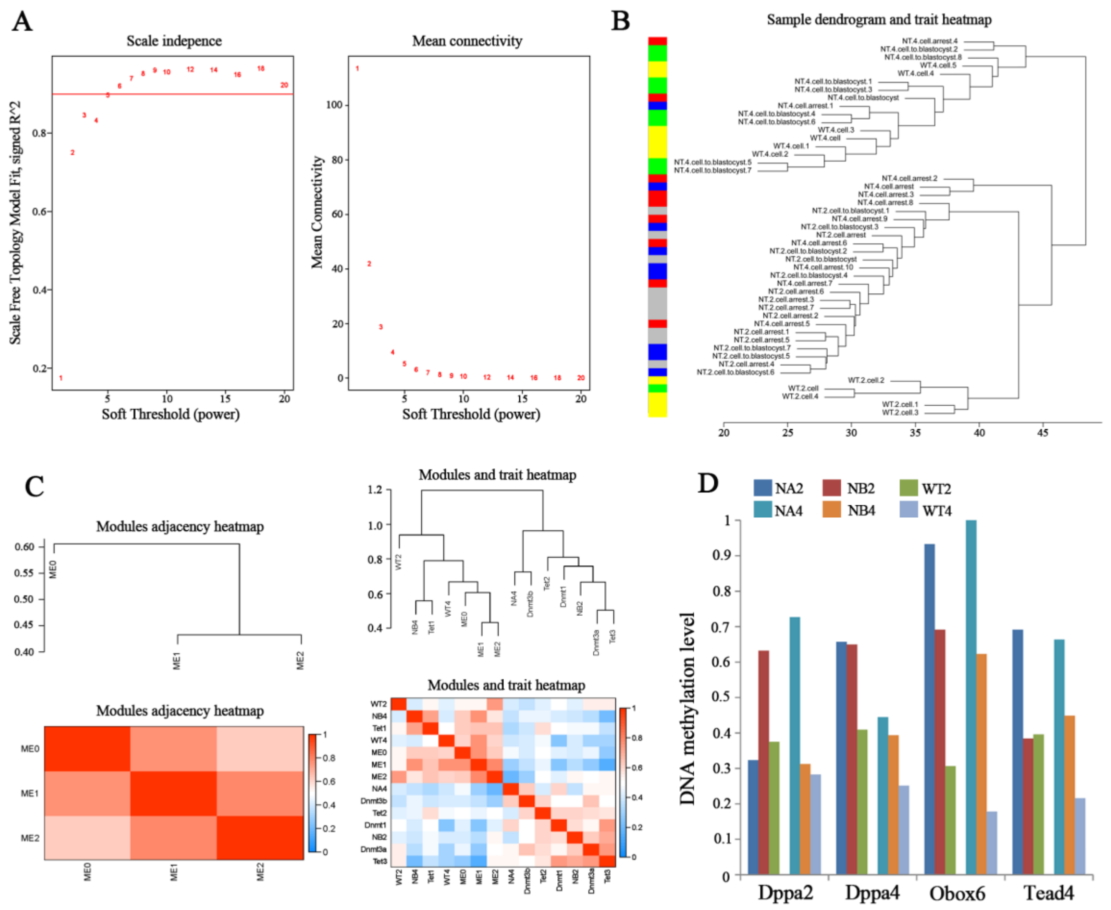

Supplementary Figure 5 Weighted gene co-expression network analysis.

(A) Threshold analysis of the weighting coefficient β in the adjacency matrix. In order to make the network close to no scale, β is selected to be 5.

(B) Sample cluster analysis. The tree view shows correlation of all samples.

(C) Module cluster analysis. Through topology matrix, three modules ME0, ME1 and ME2 were generated in hierarchical clustering (left). The methyltransferases (Dnmt1, Dnmt3a and Dnmt3b) and the demethylases (Tet1, Tet2 and Tet3) were clustered with the modules (right). Degree of correlation was indicated by colors (from blue to red).

(D) The methylation levels of four key genes (Dppa2, Dppa4, Obox6, Tead4) in SCNT embryos and *in vivo* fertilized embryos.

Supplementary Table 1 DNA methylation data set

Embryo type	Sample type	CpG number	Mean depth
SCNT	Zygote	20655983	23.13
SCNT	2-cell arrest	16960785	24.62
SCNT	2-cell to blast	16167658	28.53
SCNT	4-cell arrest	16661565	29.44
SCNT	4-cell to blast	16321695	29.09
WT	Cumulus cell	19481721	11.40
WT	Zygote	18177526	16.82
WT	2-cell	19357343	11.81
WT	4-cell	19333182	13.80

Supplementary Table 2 Transcriptome data set

Embryo type	Sample type	Sample number
SCNT	2-cell arrest	8
SCNT	2-cell to blast	9
SCNT	4-cell arrest	5
SCNT	4-cell to blast	9
WT	Cumulus cell	6
WT	MII oocyte	6
WT	Zygote	5
WT	2-cell	5
WT	4-cell	6
